# Immunohistochemical Insights into Enhanced Burn Wound Healing by Poly(vinyl alcohol)/Gentamicin and Poly(vinyl alcohol)/Chitosan/Gentamicin Hydrogels

**DOI:** 10.3390/gels12100925

**Published:** 2026-10-14

**Authors:** Anja Nikolić, Ivan Milošević, Ana Janković, Bogomir Bolka Prokić, Emilija Nićković, Milena Stevanović, Marija Majstorović, Milorad Mirilović, Vesna Mišković-Stanković, Tijana Lužajić Božinovski

**Affiliations:** 1 Department of Histology and Embryology, Faculty of Veterinary Medicine, University of Belgrade, Bulevar Oslobodjenja 18, 11000 Belgrade, Serbia; anja.nikolic@vet.bg.ac.rs (A.N.); nickovic.emilija@vet.bg.ac.rs (E.N.); majstorovic.marija@vet.bg.ac.rs (M.M.); ticavet@vet.bg.ac.rs (T.L.B.); 2 Innovation Center of the Faculty of Technology and Metallurgy, University of Belgrade, Karnegijeva 4, 11000 Belgrade, Serbia; ajankovic@tmf.bg.ac.rs (A.J.); mstevanovic@tmf.bg.ac.rs (M.S.); 3 Department of Surgery, Orthopedy and Ophthalmology, Faculty of Veterinary Medicine, University of Belgrade, Bulevar Oslobodjenja 18, 11000 Belgrade, Serbia; bbprokic@gmail.com; 4 Department of Economics and Statistics, Faculty of Veterinary Medicine, University of Belgrade, Bulevar Oslobodjenja 18, 11000 Belgrade, Serbia; mija@vet.bg.ac.rs; 5 Faculty of Ecology and Environmental Protection, University Union—Nikola Tesla, Cara Dušana 62–64, 11000 Belgrade, Serbia; vesna.miskovicstankovic@unionnikolatesla.edu.rs

**Keywords:** angiogenesis, cell proliferation, macrophages, skin regeneration, tissue remodeling

## Abstract

Novel poly (vinyl alcohol) (PVA)/gentamicin (Gent) and PVA/chitosan (CHI)/Gent hydrogels have previously demonstrated improved burn wound healing in a rat model, based on clinical assessment and histological analysis. This study aims to provide immunohistochemical insight into the effects of PVA/Gent and PVA/CHI/Gent on burn wound healing by analyzing markers of macrophage presence (CD68), angiogenesis (VEGF and ESM1), proliferation (Ki-67), and apoptosis (TUNEL) on post-burn days 3, 7, 14 and 21. Both hydrogels increased macrophage presence, with PVA/CHI/Gent producing earlier effects. Angiogenesis was more pronounced in the PVA/CHI/Gent-treated group, in terms of both the number of VEGF^+^ cells and the relative density of blood vessels (ESM1), while PVA/Gent generally exceeded the control values without significant differences. On day 21, macrophage presence and angiogenesis were reduced in the treated groups, with significantly higher values in the control group. Proliferation was greater in the treated groups during the early healing phase (on days 3 and 7), whereas it was predominant in the control group during the later stages (on days 14 and 21). Conversely, apoptosis was predominant in the control group at the first three time points; however, by day 21, it was more pronounced in the treated groups, particularly in the PVA/CHI/Gent-treated group. Overall, both hydrogels showed similar temporal cellular and tissue responses, but PVA/CHI/Gent induced earlier and more sustained effects and demonstrated superior pro-angiogenic activity.

## 1. Introduction

Burn wounds are the cause of approximately 180 000 deaths annually according to the WHO, with the highest rates of mortality and disability reported in developing countries [1]. Additionally, burns are also classified as chronic wounds, characterized by the impaired process of wound healing due to a multitude of factors [2]. The estimated prevalence of chronic wounds with a mixed etiology in the general population is 1–2%, which is similar to the estimated prevalence of cardiovascular system diseases [3]. Considering the above-mentioned facts, the development and application of affordable dressings that have the ability to normalize the healing process of burn wounds presents as an urgent task for public health [2]. An ideal wound dressing should ensure a moist wound environment while absorbing excess exudate, allow for gaseous exchange, protect the wound from microorganisms, promote tissue regeneration, enable the mechanical support of the wound while possessing elasticity to adapt to movement, possess biocompatibility and biodegradability without creating toxic by-products, and be widely available and affordable [4]. Considering that the concept of sustainability is becoming a cornerstone of research across diverse scientific fields, focusing on characteristics of sustainability and eco-friendliness in the development of wound dressings is becoming more prevalent [4]. Hydrogel-based wound dressings meet many of the criteria that define ideal dressings, and are thus considered one of the most promising materials used in wound care, which is supported by the fact that hydrogels occupy most of the global market for dressings [5,6]. The unique properties of hydrogels, such as their high porosity, swelling ability, biocompatibility and biodegradability, underlie their high potential to be used as systems for the targeted delivery of drugs, antibiotics, nanoparticles, regulatory peptides and growth factors [2,7,8]. The possibility of adjusting hydrogel formulations by adding different cross-linking agents, metal ions, nanoenzymes and substances capable of responding to diverse conditions has led to the designing of hydrogel dressings with multiple application forms and functions, the ability to respond to stimuli, and that enable intelligent release of active substances, which position hydrogels as the most competitive wound dressings [9]. New studies have described the designing of hydrogel dressings aimed for use in specific scenarios, such as shape-adaptive bilayer hydrogels, created for individuals carrying out field operations in extreme conditions, to treat irregular wound that cannot be compressed and to offer protection from insect bites and possible secondary infections [10]. The novel hydrogel formulations of poly (vinyl alcohol) (PVA)/gentamicin (Gent) and PVA/chitosan (CHI)/Gent, which are the subject of this study, were designed to be used as dressings for medical treatment of deep chronic wounds [11]. Both the PVA and CHI polymers used in hydrogel synthesis are widely used in biomedical research and are known for their good mechanical properties; excellent biocompatibility and safety; and good cost effectiveness, availability and eco-friendly characteristics [4]. The choice of Gent, an antimicrobial substance, was based once again on affordability and accessibility, and the fact that this broad-spectrum antibiotic is effective against both Gram-positive and Gram-negative bacteria and, most importantly, against the bacteria that cause nosocomial infections [12]. Combining these materials’ characteristics with a relatively simple synthesis protocol provides a simple wound dressing that can be easily produced and is cost-effective, affordable and eco-friendly, which is important for the often-prolonged treatment associated with chronic wounds. Both hydrogel formulations have shown good mechanical [13], physicochemical and biological properties in terms of antibacterial activity and absence of cytotoxicity in vitro [11], as well as beneficial effects on wound healing through stimulation of re-epithelization and dermal repair in vivo [14]. Most studies of hydrogel dressings have focused on assessing the acceleration rate of burn wound healing, although acceleration by itself may lead to disorders during the tissue regeneration process [15]. It is much more beneficial to focus on assessing hydrogels in the framework of different phases of burn wound healing and developing functional hydrogels according to the different states of burn wounds, which will enable the administration of appropriate therapy at appropriate time [15].

The process of burn wound healing is a highly coordinated and dynamic process, consisting of four somewhat overlapping and associated phases: hemostasis, inflammation, proliferation and remodeling [16]. The phase of hemostasis is often disregarded in the process of burn wound healing, due to the absence of bleeding, with increased vascular permeability leading to plasma loss being present instead [1]. Cells of different skin lineages contribute to normal tissue regeneration, and their behavior is regulated by different mechanical and chemical signals, most of which originate from inflammatory cells that are attracted to the wound bed by damage signals and signals from the pathogens [17]. The inflammatory phase thus not only enables the cleaning of debris and potentially present pathogens from the wound, but is considered as the beginning of tissue repair and regeneration [18,19]. The two most prominent cell types present during this phase are neutrophils, which represent the primary effector cells in the initiation of inflammation, and macrophages, which appear subsequently and soon become the dominant population of immune cells in the wound bed [18]. Macrophages are present in all phases of wound healing, and have roles in inflammation and resolving inflammation, affecting the proliferative processes and initiating and maintaining remodeling [18,20]. Great plasticity is a well-known attribute of macrophages, allowing them to differentiate into different phenotypes or even transdifferentiate into different cell types depending on the wound microenvironment [21,22]. The differentiation of macrophages into different phenotypes during wound healing is used in forensic studies exploring wound age determination [23]. Dysfunctionality of macrophages is recognized as one of the key factors in delayed healing of wounds associated with aging and diabetes, as well as the excessive healing associated with fibrosis [18,24]. Considering the importance of macrophages across all phases of wound healing, these cells are recognized as promising therapeutic targets for the regulation of the healing process [18]. Timely transition from the inflammatory to the proliferative phase is an essential step for successful wound healing [25]. The transition is regulated by many different factors, including bioactive lipid mediators; reactive oxygen species; genome expression regulators; and cellular factors like macrophages, which are also recognized as a regulator of the proliferative phase [18,26]. As the name suggests, the proliferative phase is characterized by proliferation of many different types of cells (fibroblasts, keratinocytes and endothelial cells), which enables the unfolding of intertwined processes that define this phase: fibroplasia, angiogenesis and re-epithelization [18]. Angiogenesis is a key process in tissue regeneration, during which the density of the newly formed capillary network may exceed that of normal tissue by threefold or more, thereby providing the oxygen and nutrients necessary for the proliferation, migration and metabolic activity of cells participating in tissue repair [27,28]. Many pro-angiogenic molecules (growth factors and cytokines) are secreted during the inflammatory phase to enable angiogenesis; out of them, VEGF is the most potent and is considered the prime regulator of wound healing angiogenesis [27,29]. Chronic wounds are often characterized by extremely low levels of VEGF, which is likely associated with impaired vasculature and wound healing [30]. Wound-edge keratinocytes and macrophages are the most prominent sources of VEGF, but studies have shown that fibroblasts and mast cells of injured skin also express VEGF [17,31]. The extra-angiogenic effects of VEGF in wound healing; the regulation of proliferation, migration and survival of keratinocytes; and scar formation are also important topics in wound healing research [31,32]. The end result of proliferative phase is formation of premature granulation tissue characterized by a dense capillary network, high numbers of fibroblasts and macrophages and irregularly arranged collagen bundles [33]. The last phase of wound healing, remodeling, can progress for even a year after injury and is characterized by progressive loss of cellularity and vasculature [33]. The process of cell apoptosis is responsible for stopping the formation of granulation tissue and, through the removal of inflammatory cells, apoptosis enables the evolution of granulation tissue into scar tissue, which is the physiological endpoint of mammalian wound healing [34,35]. Angiogenic processes diminish in parallel, leading to declined blood flow to the wound and consequently the slowing and final stopping of the wound’s metabolic activity [34]. Phase of remodeling is also characterized by extensive changes in the ECM, primarily due to the gradual replacement of collagen type III, characterized by thin, elastic fibers deposited during the early stages of healing, with collagen type I, which is characterized by thick, densely packed fibers that provide high tensile strength [36].

The aim of this study was to characterize the temporal cellular and tissue responses associated with the beneficial effects of PVA/Gent and PVA/CHI/Gent hydrogels on burn wound healing shown in our previous study [14], by analyzing immunohistochemical markers of macrophage presence (CD68), angiogenic activity (VEGF and ESM1), cell proliferation (Ki-67), and apoptosis (TUNEL) across different phases of the healing process.

## 2. Results and Discussion

**Figure 1:**
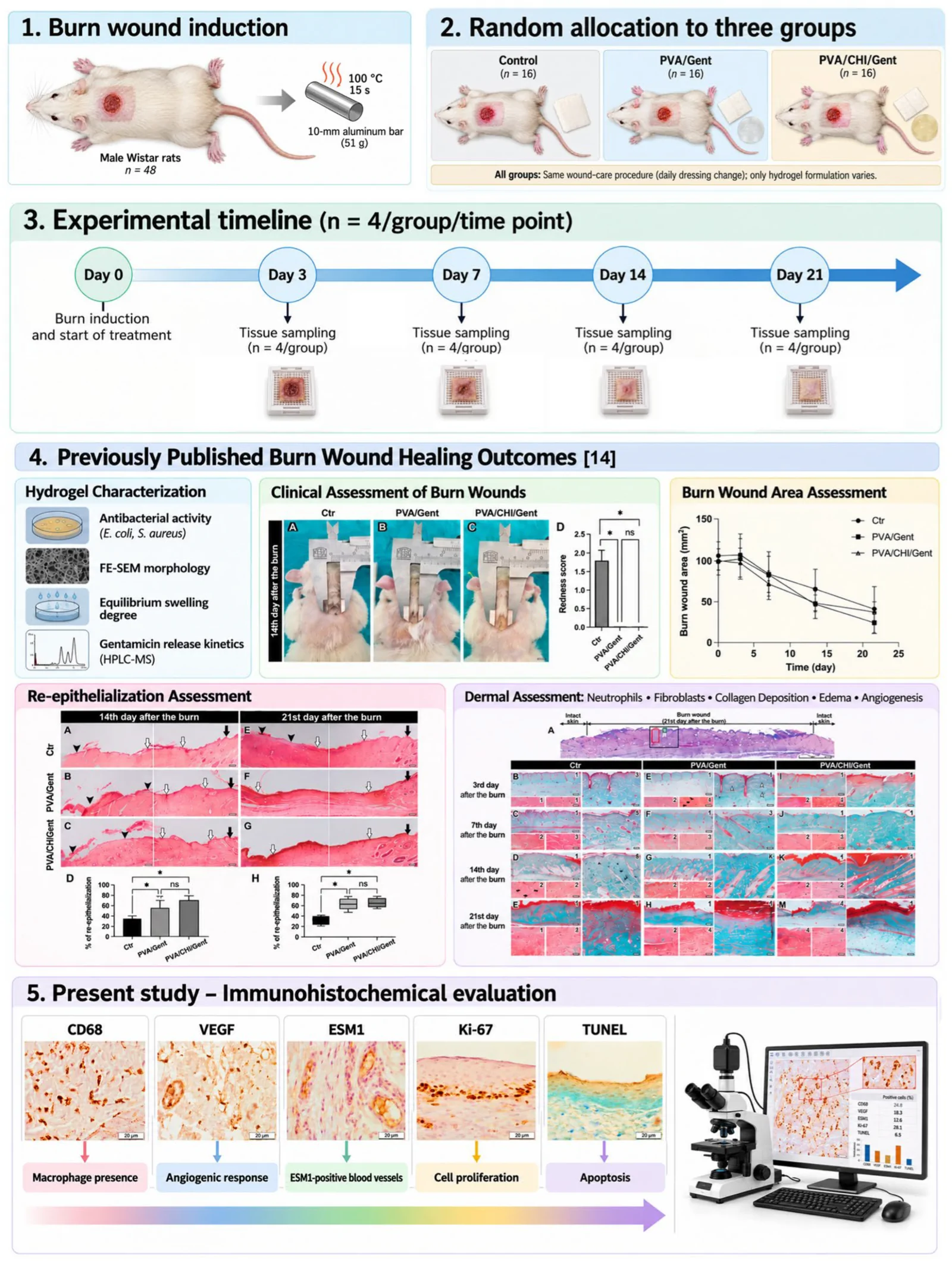
Schematic overview of the experimental design and study workflow. Previously published wound healing outcomes presented in the subfigures of part 4 of Figure 1 are adapted from [14]. Selected schematic elements were generated using ChatGPT (OpenAI, San Francisco, CA, USA), subsequently modified using Codex (GPT-6; OpenAI), and edited and assembled using Adobe Photoshop (Adobe Inc., San Jose, CA, USA).

This study examined tissue samples obtained from a previously established rat burn wound model [37] comprising three experimental groups: Ctr, PVA/Gent-treated, and PVA/CHI/Gent-treated group. Following standardized thermal injury induction and group allocation, the wounds were treated according to the experimental protocol: macroscopic assessment of clinical parameters, wound area measurements and tissue samples were collected on post-burn days 3, 7, 14, and 21. The results of clinical assessments, wound area measurements, and the histological analysis conducted on sections stained with HE and Mason Goldner staining, which included the determination of percentage of re-epithelization and semi-quantitative assessment of the dermis, were presented in our previous study [14]. Our previous research [14] showed that both hydrogel formulations significantly reduced inflammation, enhanced re-epithelization and increased fibroblast proliferation and collagen deposition, thus showing potential as effective wound dressings. In our present study, immunohistochemical analyses were performed to enable better understanding of biological processes and their temporal pattern during wound healing under the influence of novel hydrogels. For this purpose, the macrophage presence (CD68), angiogenic activity (VEGF and ESM1), cell proliferation (Ki-67), and apoptosis (TUNEL) were examined. A schematic overview of the experimental design and both the previous and present study is presented in Figure 1.

### 2.1. Macrophage (CD68 Positive) Presence and Distribution in the Dermis of Burn Wounds

**Figure 2:**
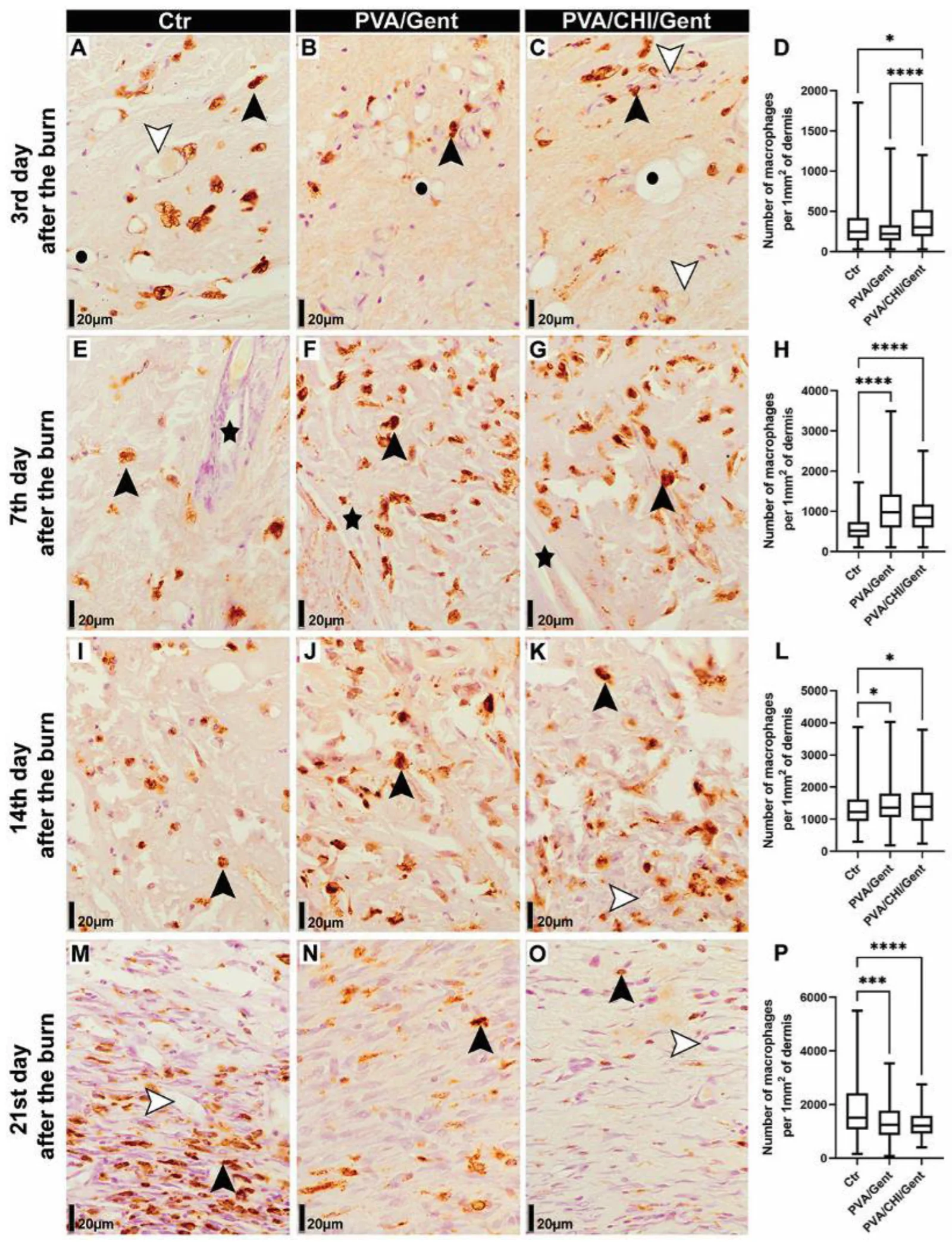
Temporal changes in CD68^+^ macrophages during burn wound healing. Representative images of CD68-immunostained sections counterstained with Mayer’s hematoxylin and viewed with high-power objective lens (40×) from control (Ctr), PVA/Gent, and PVA/CHI/Gent groups on days 3 (**A**–**C**), 7 (**E**–**G**), 14 (**I**–**K**), and 21 (**M**–**O**) after burn induction. Black arrowheads indicate CD68^+^ macrophages, white arrowheads indicate blood vessels, black stars indicate hair follicles, and black circles indicate adipocytes; scale bar = 20 μm. Quantification of CD68^+^ macrophages in dermis is shown for days 3 (**D**), 7 (**H**), 14 (**L**), and 21 (**P**). Data are presented as median, interquartile range, and minimum–maximum values. * *p* < 0.05, *** *p* < 0.001, **** *p* < 0.0001.

On day three post-burn induction, CD68^+^ macrophages were predominantly distributed in the deeper dermal layers, perivascular and interstitial, between adipocytes. The highest density was observed in the PVA/CHI/Gent-treated wounds, significantly higher than in the control and PVA/Gent-treated groups (*p* < 0.05 and *p* < 0.0001, respectively). In contrast, the PVA/Gent group showed a non-significant moderate decrease relative to the control (Figure 2A–D). The dominant presence of macrophages in the PVA/CHI/Gent-treated group was expected considering that one of its components, CHI, is attributed to have immunomodulatory properties and can enhance macrophage recruitment, establish a pro-regenerative microenvironment, and improve burn wound healing [38,39]. The influence of CHI can be explained by degradation of hydrogels on the surface of a wound. Being placed on a wound’s surface, hydrogels are affected by the different physical and chemical conditions in terms of temperature, chemical microenvironment and, most importantly, pH. A wound’s surface layer pH is around 7.4 or higher [40], due to the exposure of underlaying tissue, and CHI is especially sensitive in neutral and basic ranges of pH. Fully formed chitosan-based or composite hydrogels subjected to alkaline/basic pH ranges often undergo structural loosening, compaction changes, or polymer relaxation. Therefore, the conditions of our study led to the degradation of composite hydrogel containing CHI, which enabled the CHI to interact with the burn wound and subsequently caused the changes to the PVA/CHI polymeric matrix in the burn microenvironment.

In addition to the phagocytic activity, macrophages are key cells in wound healing and orchestrate the switch from inflammation to regeneration by secreting cytokines and growth factors that stimulate various processes associated with repair [41,42]. By day 7, macrophages had spread into the superficial dermis around the damaged hair follicles and sebaceous glands. The two treated groups contained significantly higher numbers of CD68^+^ cells than the control (*p* < 0.0001), while no significant difference was observed between the two hydrogel formulations (Figure 2E–H). This continuous presence of macrophages coincided with the onset of the proliferative phase, during which they activated fibroblasts and stimulated extracellular matrix (ECM) deposition and angiogenesis via the release of mediators, such as VEGF, TGF-β, and PDGF [43,44]. Thus, the earlier and more intense macrophage recruitment in the treated wounds could have contributed to the faster healing response seen in both hydrogel groups [14]. Similar effects were reported for bioactive wound dressings that modulate the inflammatory response and allow for timely progression to tissue regeneration [38]. On day 14, this trend continued, and the number of macrophages in both hydrogel-treated groups remained significantly higher than in the control group (*p* < 0.05) (Figure 2I–L). However, this difference was less pronounced, as macrophage infiltration had also increased in the untreated wounds. The delayed rise in the control group may indicate protracted inflammation. Effective wound healing does not depend only on the number of macrophages but also on the appropriate timing of their functional transition. Early recruitment and resolution of inflammation are generally associated with better healing outcomes [22,42]. By day 21, this trend was reversed, with the control group having significantly more macrophages than the PVA/Gent- and PVA/CHI/Gent-treated groups (*p* < 0.001 and *p* < 0.0001, respectively), while no significant difference was observed between the two treated groups (Figure 2M–P). Under physiological conditions, the number of macrophages declines with the onset of tissue remodeling. On the other hand, the persistent infiltration of macrophages in the untreated wounds corresponds to a delayed healing process and active inflammation. Studies have shown that prolonged macrophage presence is a feature of delayed or chronic wound healing, whereas successful repair is associated with early but transient macrophage presence [41,45].

Since CD68 is a pan-macrophage marker, the present study did not differentiate between pro-inflammatory and reparative macrophage phenotypes. A shifted temporal pattern of macrophage presence characterized by earlier increase followed by a decline in CD68^+^ cells was evident in the hydrogel-treated wounds when compared to the Ctr group wounds. Based on the current immunohistochemical analysis, we can only speculate that this pattern could reflect an earlier resolution of inflammation, but definitive confirmation of this claim requires further research on macrophage phenotypization to be conducted.

### 2.2. Assessment of the Angiogenesis Process in the Burn Wound Dermis

**Figure 3:**
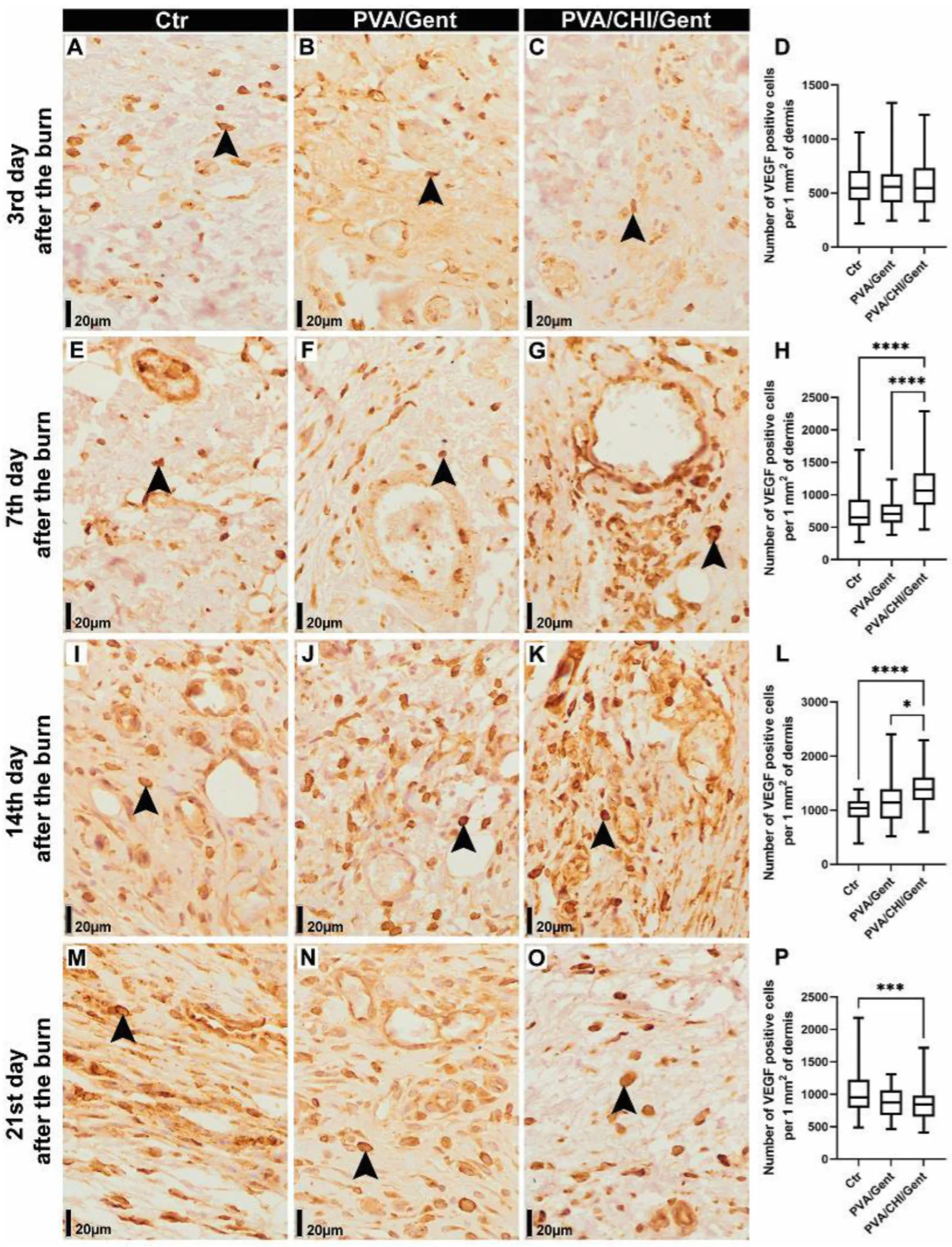
Temporal changes in VEGF expression during burn wound healing. Representative images of VEGF-immunostained sections counterstained with Mayer’s hematoxylin and viewed with a high-power objective lens (40×) from the control (Ctr), PVA/Gent, and PVA/CHI/Gent groups on days 3 (**A**–**C**), 7 (**E**–**G**), 14 (**I**–**K**), and 21 (**M**–**O**) after burn induction. Black arrowheads indicate VEGF+ cells; scale bar = 20 μm. The number of VEGF+ cells per 1 mm2 of dermis is shown for days 3 (**D**), 7 (**H**), 14 (**L**), and 21 (P). Data are presented as median, interquartile range, and minimum–maximum values. * *p* < 0.05, *** *p* < 0.001, **** *p* < 0.0001.

Angiogenesis in the burn wound dermis was evaluated by VEGF expression and relative density of ESM1^+^ blood vessels. These two complementary markers reflect different stages of the angiogenic process, with VEGF representing an early molecular trigger of neovascularization and ESM1 representing the activated endothelial cells of the newly formed micro vessels [31,46]. On day 3, both hydrogels showed increased numbers of VEGF^+^ cells compared with the control, although the increase was not significant (Figure 3A–D). However, these minor increases indicate that both hydrogels can activate angiogenic signaling shortly after injury. Rapid upregulation of VEGF in response to tissue hypoxia and inflammatory stimuli is an important mediator of endothelial cell proliferation, migration, and capillary formation in wound healing [47,48]. On day 7, a marked increase in VEGF expression was seen in the PVA/CHI/Gent group, which showed significantly higher numbers of VEGF^+^ cells than the control and PVA/Gent groups (*p* < 0.0001), while the increase in the PVA/Gent-treated group was not significant (Figure 3E–H). This pattern persisted on day 14, with the PVA/CHI/Gent hydrogel again showing significantly higher VEGF expression than both the control and PVA/Gent-treated groups (*p* < 0.0001 and *p* < 0.05, respectively) (Figure 3I–L). At the same time point, the increase in VEGF expression was also evident in the PVA/Gent-treated group, although without significance (Figure 3I,J,L). Hence, the composite hydrogel containing CHI showed markedly enhanced pro-angiogenic activity. Previous studies have shown that CHI-based biomaterials promote VEGF secretion and vascular regeneration by creating a supportive microenvironment for endothelial and reparative immune cells [49,50]. Interestingly, the temporal pattern of VEGF expression mirrored the macrophage response observed in our study. Since macrophages are one of the major cellular sources of VEGF during wound healing, their earlier recruitment in the PVA/CHI/Gent-treated wounds may have contributed to the higher VEGF expression detected during the proliferative phase [41,44]. On day 21, VEGF expression was downregulated in both hydrogel-treated groups but was still increasing in the untreated wounds; hence, VEGF expression was significantly higher in the control group than in the PVA/CHI/Gent-treated group (*p* < 0.001) (Figure 3M–P). This occurrence does not necessarily suggest a deficit in angiogenic potential but more likely reflects differences in the kinetics of healing. Angiogenesis is a transient event peaking during granulation tissue formation and declining with vascular maturation and tissue remodeling [31]. Sustained VEGF expression is associated with chronic inflammation and delayed wound healing [31].

**Figure 4:**
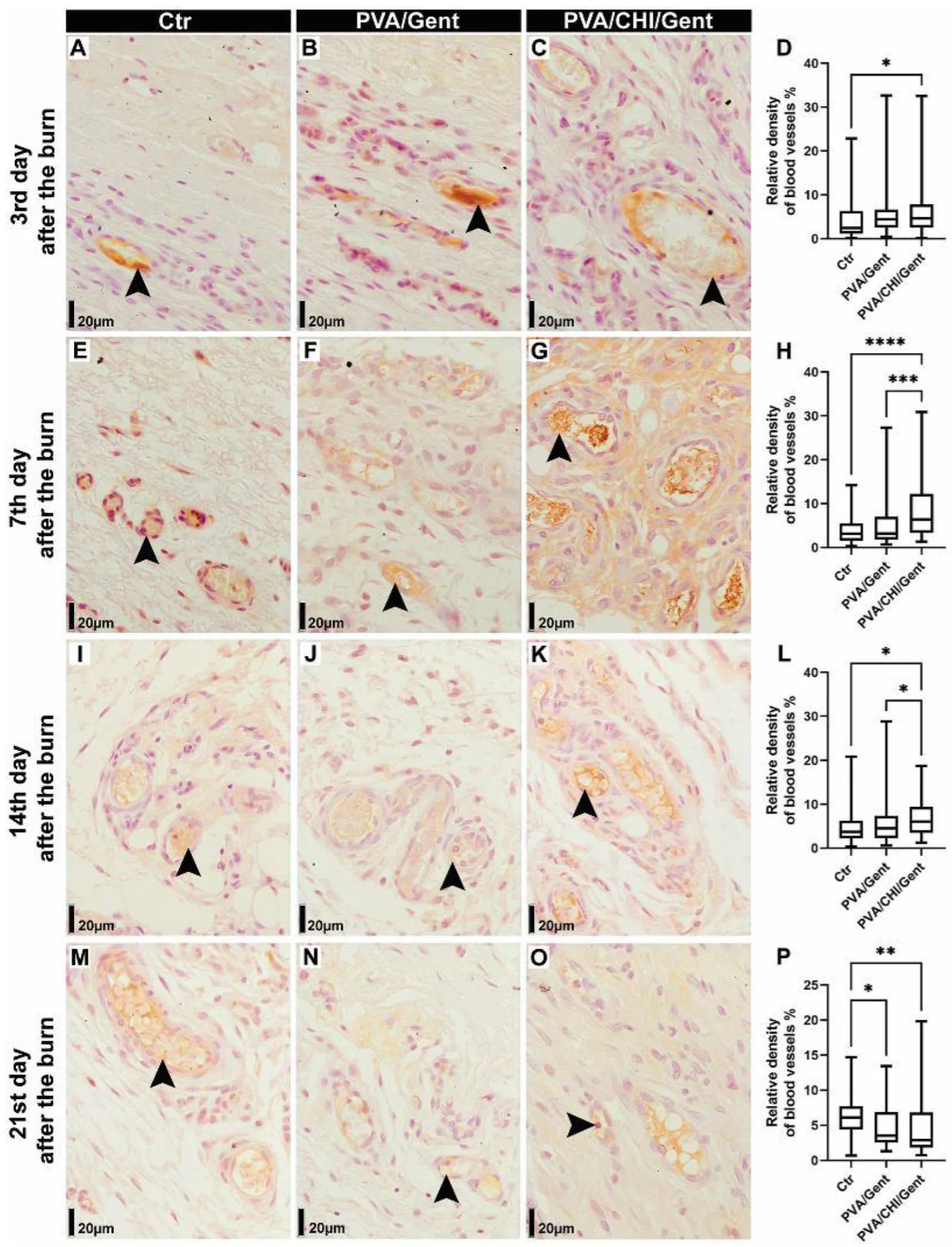
Temporal changes in ESM1-positive blood vessels during burn wound healing. Representative images of ESM1-immunostained sections counterstained with Mayer’s hematoxylin and viewed with a high-power objective lens (40×) from the control (Ctr), PVA/Gent, and PVA/CHI/Gent groups on days 3 (**A**–**C**), 7 (**E**–**G**), 14 (**I**–**K**), and 21 (**M**–**O**) after burn induction. Black arrowheads indicate ESM1^+^ blood vessels; scale bar = 20 μm. The relative density of blood vessels in the dermis is shown for days 3 (**D**), 7 (**H**), 14 (**L**), and 21 (**P**). Data are presented as median, interquartile range, and minimum–maximum values. * *p* < 0.05, ** *p* < 0.01, *** *p* < 0.001, **** *p* < 0.0001.

The presence of ESM1^+^ vessels corroborates the VEGF findings. On day 3, the vascular density was higher in both treated groups than in the control, achieving significance only in the PVA/CHI/Gent group (*p* < 0.05) (Figure 4A–D). On both days 7 and 14, the vascular density increased in all experimental groups, but the increase was still only significant in the PVA/CHI/Gent-treated wounds compared to the control and PVA/Gent-treated group (day 7, *p* < 0.0001 and *p* < 0.001, respectively; day 14, *p* < 0.05) (Figure 4E–L). The presence of ESM1-positive vascular structures supports the VEGF findings by providing morphological evidence of endothelial activation associated with molecular activation [51,52].

By day 21, the vascular density declined in both hydrogel-treated groups but continued to increase in the untreated wounds, with the vessel density being significantly higher in the control group compared to the PVA/Gent- and PVA/CHI/Gent-treated groups (*p* < 0.05 and *p* < 0.01, respectively) (Figure 4M–P). The pattern of vascular density decreasing in the treated groups and significantly increasing in the Ctr group is in accordance with the VEGF results from the same time period. Considering that persistent angiogenesis is typically associated with delayed wound maturation and chronic inflammation [44,48], this result likely does not indicate insufficient angiogenic activity of examined hydrogels.

Although both hydrogel formulations enhanced angiogenesis compared to the untreated wounds, the PVA/CHI/Gent hydrogel had a remarkably higher impact on both the VEGF expression and neovascularization, which could be linked with the good angiogenic potential associated with CHI incorporation into hydrogels [50]. The early elevation of angiogenic activity followed by its physiological decline suggests that the PVA/CHI/Gent hydrogel may have accelerated vascular regeneration without prolonging the angiogenic phase. Thus, the improved wound healing observed with this formulation might be explained by its ability to induce timely and appropriately coordinated angiogenesis.

It is important to note that VEGF and ESM1 markers do not provide information regarding pericyte recruitment or subsequent stabilization and maturation of newly formed vessels. Therefore, the present findings demonstrate enhanced angiogenic activity, but cannot establish the characteristics of newly formed blood vessels in terms of maturity or full functionality, which requires further research and use of markers, such as alpha-smooth muscle actin (αSMA).

### 2.3. Proliferation and Apoptosis in the Dermis of Burn Wounds

**Figure 5:**
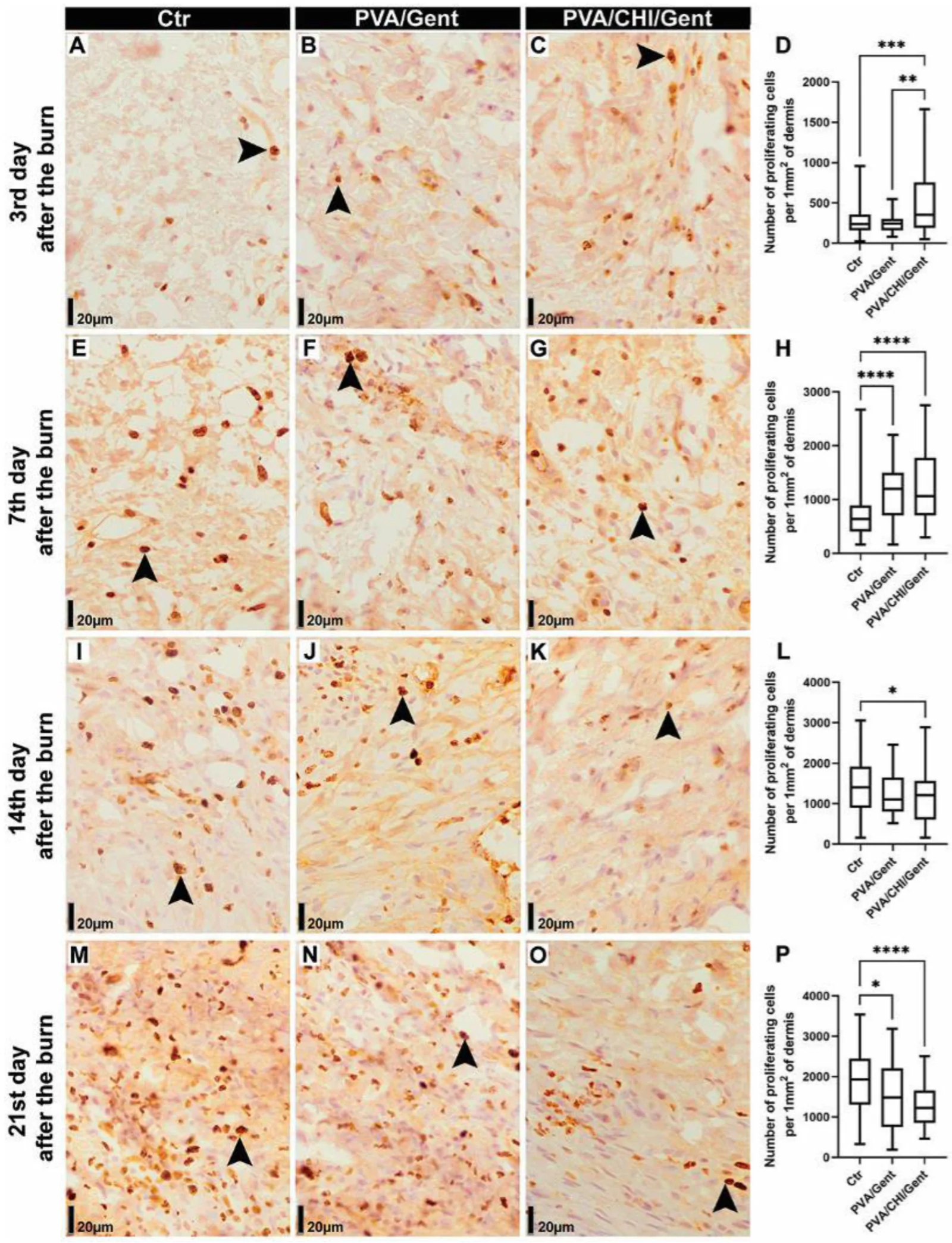
Temporal changes in dermal cell proliferation during burn wound healing. Representative images of Ki-67-immunostained sections counterstained with Mayer’s hematoxylin and viewed with a high-power objective lens (40×) from the control (Ctr), PVA/Gent, and PVA/CHI/Gent groups on days 3 (**A**–**C**), 7 (**E**–**G**), 14 (**I**–**K**), and 21 (**M**–**O**) after burn induction. Black arrowheads indicate Ki-67^+^ cells; scale bar = 20 μm. The number of proliferating cells per 1 mm^2^ of dermis is shown for days 3 (**D**), 7 (**H**), 14 (**L**), and 21 (**P**). Data are presented as median, interquartile range, and minimum–maximum values. * *p* < 0.05, ** *p* < 0.01, *** *p* < 0.001, **** *p* < 0.0001.

Ki-67 immunohistochemistry was applied to evaluate cell proliferation within the burn wound dermis. On day 3, during the early inflammatory phase, significantly higher numbers of Ki-67^+^ cells were observed in the PVA/CHI/Gent-treated group compared to the control and PVA/Gent groups (*p* < 0.001 and *p* < 0.01, respectively), while no difference was observed between the control and PVA/Gent-treated group (Figure 5A–D). These data suggest that the PVA/CHI/Gent hydrogel induced earlier activation of cell proliferation, a prerequisite for the replacement of damaged dermal cells and the induction of granulation tissue formation, which is a characteristic of CHI-based hydrogels [50]. Early fibroblast, endothelial, and other stromal cell proliferation is now well established as a necessary event in good wound repair [48,53]. On day 7, the two hydrogels induced an enormous increase in proliferative activity, with both having significantly higher numbers of Ki-67^+^ cells than the control (*p* < 0.0001) (Figure 5E–H). This again mirrors the increased macrophage infiltration and enhanced angiogenesis seen over this period and suggests a coordinated progression into the proliferative healing phase. Macrophage-derived cytokines and growth factors stimulate fibroblast proliferation, and the newly established vasculature supplies oxygen and nutrients for active tissue regeneration [41,44]. However, a significant temporal shift occurred during the healing’s later stages. On both days 14 and 21, proliferative activity was higher in the untreated wounds than in either hydrogel-treated group, with significance being reached only in comparison with the PVA/CHI/Gent-treated group on day 14 (*p* < 0.05) (Figure 5I–L) and with both the PVA/Gent- and PVA/CHI/Gent-treated groups on day 21 (*p* < 0.05 and *p* < 0.001, respectively) (Figure 5M–P). While increased cell proliferation benefits the early phase of repair, its continuance into the later phase is commonly considered a sign of delayed tissue maturation rather than superior regeneration. In wounds healing typically, proliferation is reduced as ECM remodeling and scar maturation become the predominant biological processes [53]. Therefore, the reduced Ki-67 expression in the hydrogel-treated wounds at the later time points reflects earlier completion of the proliferative phase.

**Figure 6:**
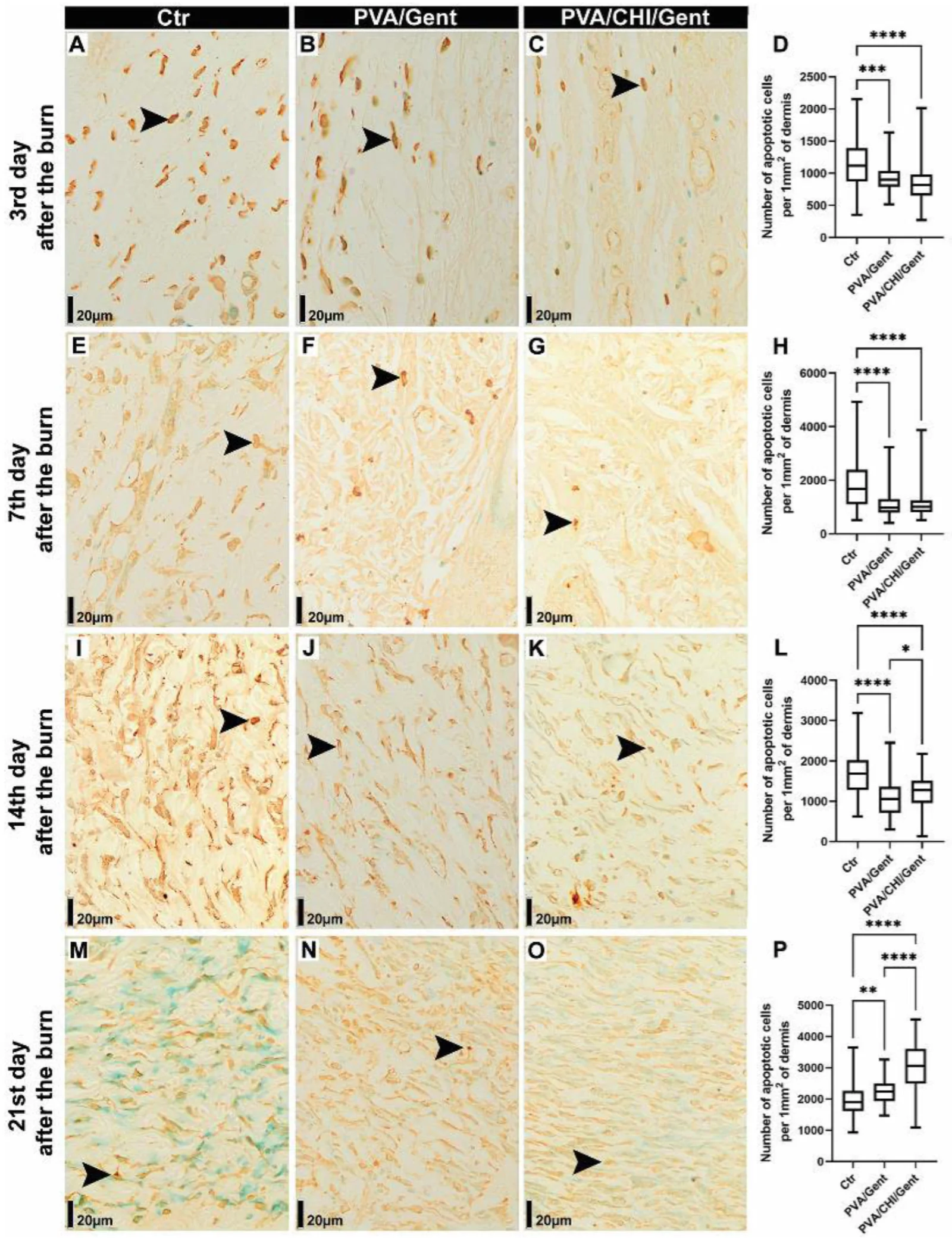
Temporal changes in dermal apoptosis during burn wound healing. Representative images of TUNEL assay-stained sections counterstained with methyl green and viewed with a high-power objective lens (40×) from the control (Ctr), PVA/Gent, and PVA/CHI/Gent groups on days 3 (**A**–**C**), 7 (**E**–**G**), 14 (**I**–**K**), and 21 (**M**–**O**) after burn induction. Black arrowheads indicate TUNEL^+^ cells; scale bar = 20 μm. The number of apoptotic cells per 1 mm^2^ of dermis is shown for days 3 (**D**), 7 (**H**), 14 (**L**), and 21 (**P**). Data are presented as median, interquartile range, and minimum–maximum values. * *p* < 0.05, ** *p* < 0.01, *** *p* < 0.001, **** *p* < 0.0001.

Dermal apoptosis, detected by a TUNEL assay, followed an opposite time course: on days 3 and 7, apoptosis was significantly higher in the untreated wounds compared to both the PVA/Gent- and PVA/CHI/Gent-treated groups (day 3, *p* < 0.001 and *p* < 0.0001, respectively; day 7, *p* < 0.001) (Figure 6A–H). Early excessive apoptosis after a burn could further jeopardize tissue viability by killing cells that could participate in repair [54]. Accordingly, the lower apoptotic activity in the treated groups indicates that both hydrogels preserved resident dermal cells during the acute inflammatory phase [54]. On day 14, apoptosis was still significantly higher in the control group compared to the PVA/Gent- and PVA/CHI/Gent-treated groups (*p* < 0.0001), but the PVA/CHI/Gent-treated wounds showed a moderate increase in apoptosis, which reached significance in comparison with the PVA/Gent-treated group (*p* < 0.05) (Figure 6I–L). However, at the end of the experimental period, both the PVA/Gent- and PVA/CHI/Gent-treated groups showed significantly higher apoptosis than the control (*p* < 0.01 and *p* < 0.0001, respectively), with the highest values being recorded in the PVA/CHI/Gent-treated group, leading to significance in comparison of the treated groups (*p* < 0.0001) (Figure 6M–P). The temporal occurrence of increased apoptosis coincided with the later stage of tissue repair, during which cellularity normally decreases as granulation tissue undergoes remodeling [54,55]. However, because the TUNEL staining was not combined with cell-type-specific markers, the identity of the apoptotic cells cannot be determined from the present analysis. Further research in terms of double staining the TUNEL assay with specific cellular makers would be beneficial to analyze exactly which cell populations undergo apoptosis and to what extent.

Thus, both hydrogel formulations accelerated cellular turnover by timely advancing the proliferative responses and granulation tissue resolution during the remodeling phase. The PVA/CHI/Gent hydrogel always induced such biological responses earlier than the PVA/Gent formulation throughout the experiment.

### 2.4. Proliferation and Apoptosis in the Newly Formed Epidermis

**Figure 7:**
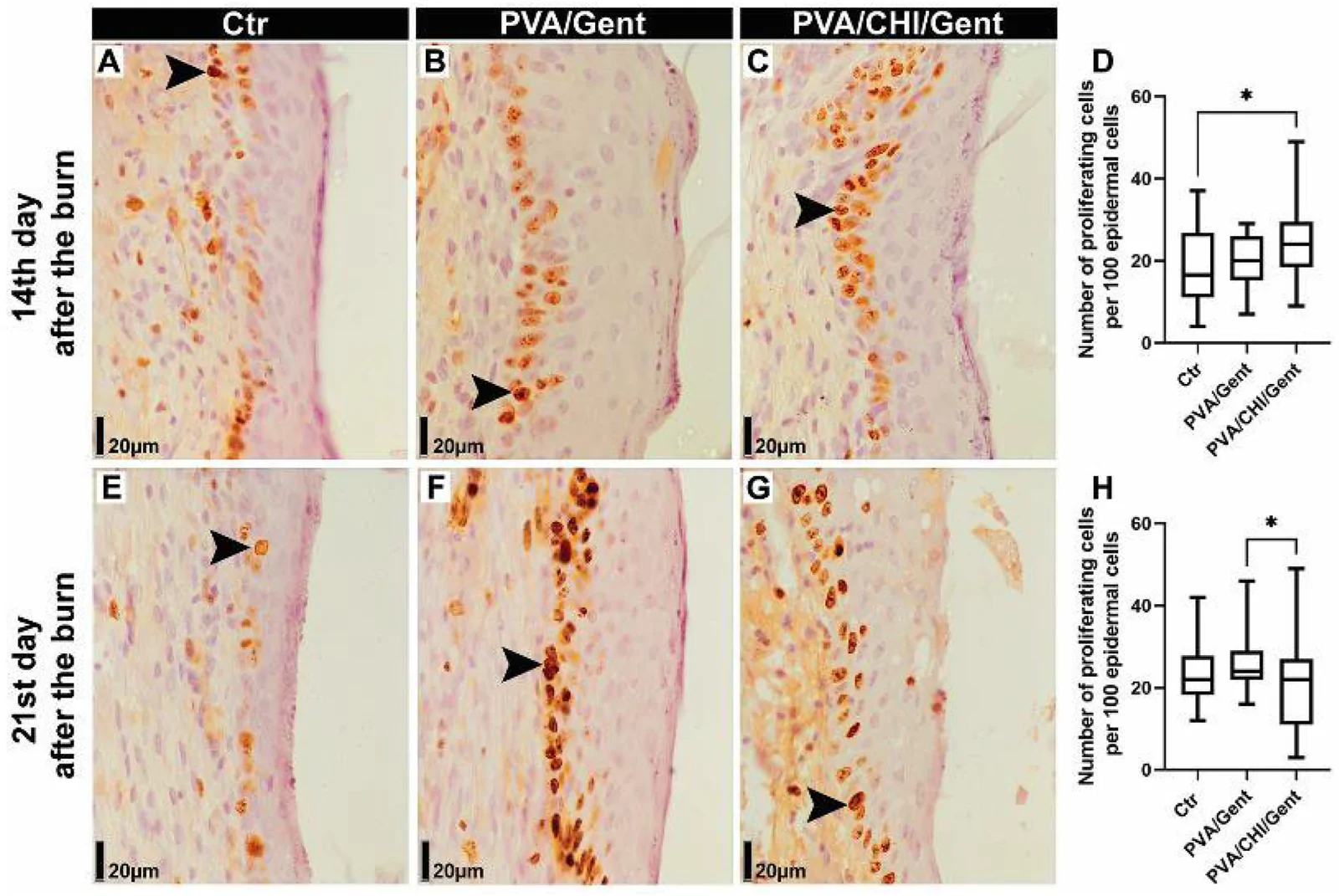
Epidermal cell proliferation during the later stages of burn wound healing. Representative images of Ki-67-immunostained sections counterstained with Mayer’s hematoxylin and viewed with a high-power objective lens (40×) from the control (Ctr), PVA/Gent, and PVA/CHI/Gent groups on days 14 (**A**–**C**) and 21 (**E**–**G**) after burn induction. Black arrowheads indicate Ki-67^+^ epidermal cells; scale bar = 20 μm. The number of proliferating cells per 100 epidermal cells is shown for days 14 (**D**) and 21 (**H**). Data are presented as median, interquartile range, and minimum–maximum values. * *p* < 0.05.

To further characterize epithelial regeneration, proliferation and apoptosis were investigated in the newly formed epidermis on days 14 and 21. On day 14, proliferation within the epidermis was higher in both hydrogel-treated groups than in the control, reaching significance only in the wounds treated with the PVA/CHI/Gent hydrogel (*p* < 0.05) (Figure 7A–D). This suggests faster re-epithelialization with the PVA/CHI/Gent hydrogel, consistent with an earlier angiogenic and proliferative response in the dermis. Adequate vascularization of the underlying granulation tissue metabolically supports keratinocyte migration and proliferation and restoration of epidermal continuity [56]. By day 21, proliferation in the epidermis further increased in the control and PVA/Gent groups, but decreased in the PVA/CHI/Gent-treated wounds, leading to Ki-67 expression being significantly lower compared to the PVA/Gent-treated group (*p* < 0.05) (Figure 7E–H). As in the dermis, this decrease in proliferative activity reflects earlier completion of epidermal regeneration and not impaired healing.

**Figure 8:**
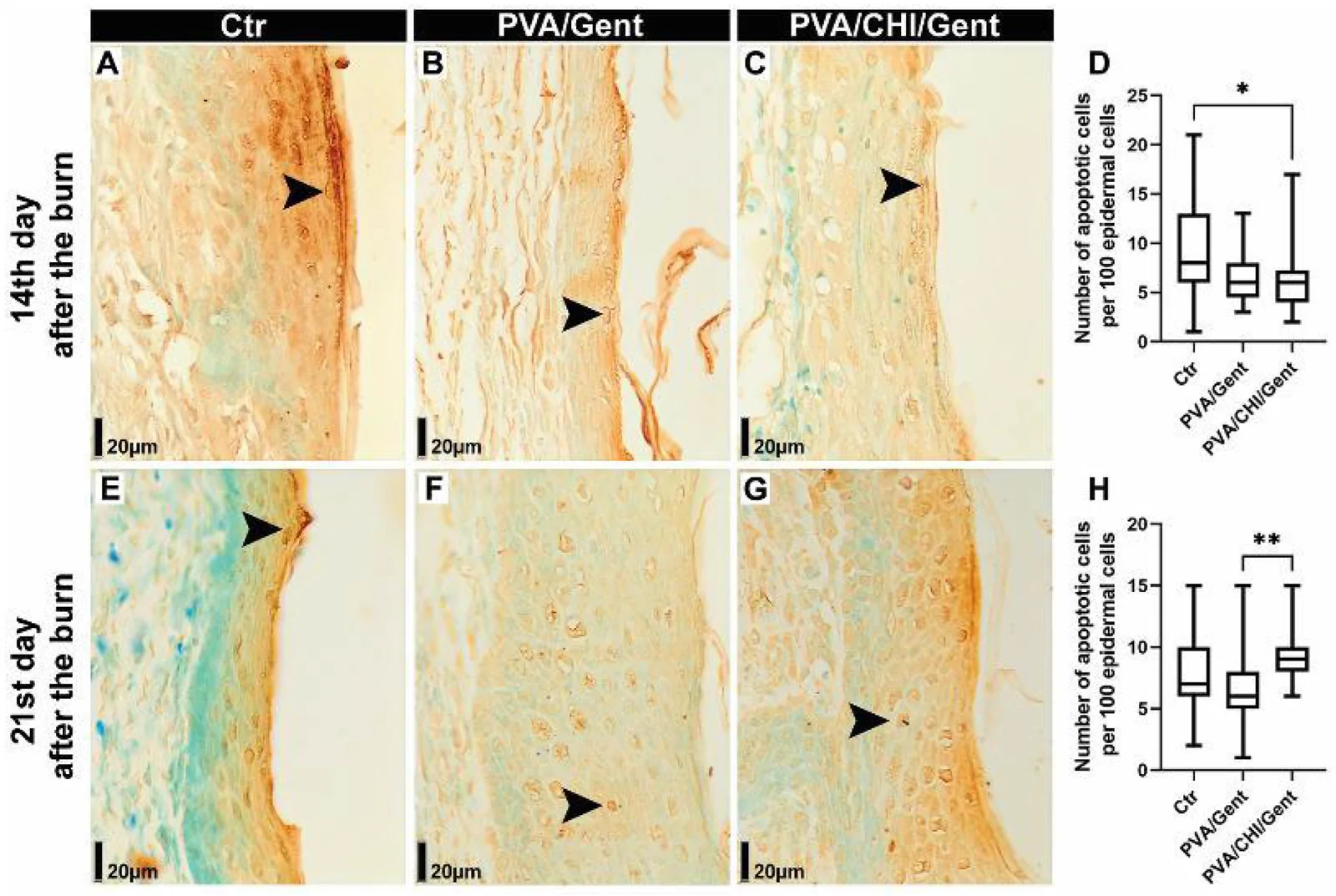
Epidermal apoptosis during the later stages of burns wound healing. Representative images of TUNEL assay-stained sections counterstained with methyl green and viewed with a high-power objective lens (40×) from the control (Ctr), PVA/Gent, and PVA/CHI/Gent groups on days 14 (**A**–**C**) and 21 (**E**–**G**) after burn induction. Black arrowheads indicate TUNEL^+^ epidermal cells; scale bar = 20 μm. The number of apoptotic cells per 100 epidermal cells is shown for days 14 (**D**) and 21 (**H**). Data are presented as median, interquartile range, and minimum–maximum values. * *p* < 0.05, ** *p* < 0.01.

The apoptosis pattern in the epidermis further supports this interpretation. On day 14, the highest level of apoptosis was noted in the control group, which was significantly higher than that found in wounds treated with the PVA/CHI/Gent hydrogels (*p* < 0.05) (Figure 8A–D). Indeed, increased keratinocyte apoptosis during active re-epithelialization has been related to delayed re-establishment of the epidermal barrier and continued tissue damage [52]. However, on day 21, the PVA/CHI/Gent-treated group had the highest apoptotic activity, significantly higher than in the PVA/Gent-treated group (*p* < 0.01) (Figure 8E–H). At this time, apoptosis probably reflects the physiological elimination of excess keratinocytes after the restoration of epidermal architecture and not continued tissue damage [57]. Similar temporal changes have been described during normal epidermal maturation, whereby apoptosis increases after wound closure to restore tissue homeostasis [54,57].

These findings in the epidermis further indicate that the PVA/CHI/Gent hydrogel accelerates re-epithelialization and promotes earlier completion of epidermal remodeling.

### 2.5. Integrated Interpretation of Immunohistochemical Analysis

When all immunohistochemical findings are interpreted in an integrated manner, a consistent temporal pattern emerges, showing that both hydrogels improved burn wound healing by synchronous enhancing of individual biological processes, which enabled the acceleration of normal tissue repair sequence. The most pronounced response in the early inflammatory phase was the enhanced accumulation of CD68^+^ macrophages, especially in the PVA/CHI/Gent-treated wounds. As central regulators of wound healing, macrophages coordinate the transition from inflammation to tissue regeneration by secreting cytokines, growth factors, and ECM remodeling enzymes [41,42]. The enhanced early macrophage recruitment coincided with increased VEGF expression and density of ESM1^+^ blood vessels, indicating accelerated angiogenesis, which could have translated into faster development of the vasculature network needed to support tissue regeneration [44]. The enhanced angiogenesis was matched by increased proliferative activity in the dermis and newly formed epidermis during the early phases of healing. The increased oxygen and nutrient supply provided through the newly formed microvasculature could have thus created an environment conducive to fibroblast expansion, ECM synthesis, and keratinocyte proliferation, leading to more rapid restoration of both the dermal and epidermal compartments [56]. Notably, this early proliferative response was transitory rather than sustained. With the further progression of healing, the proliferative activity of hydrogel-treated wounds declined earlier, with an increase in apoptosis during the last stage of the experiment, coinciding with the increased apoptotic activity expected during later stages of tissue remodeling. Taken together, the timing of macrophage infiltration, angiogenesis, and cell proliferation indicate that the hydrogels allowed the natural order of events during wound healing to proceed, although at an accelerated step from one phase into another when compared to the Ctr group. These biological events thus occurred later in the untreated wounds and persisted longer, likely due to the delayed resolution of inflammation and prolonged tissue repair. Among the formulations investigated, the PVA/CHI/Gent hydrogel produced the earliest and most marked biological responses. It is evident that the hydrogel incorporating CHI allowed for faster macrophage recruitment, more pronounced angiogenic activity, earlier cell proliferation, and a more advanced tissue remodeling stage than treatment with the hydrogel containing only PVA and Gent. This is likely a result of synergistic effects of PVA matrix providing a moist environment, and the local antibacterial effects of Gent and the biological effects of CHI in terms of their immunomodulation, antimicrobial potential and pro-regenerative action [14,50].

Overall, this would explain the superior wound healing performance achieved with the PVA/Gent and, particularly, the PVA/CHI/Gent hydrogels due to their better recruitment of macrophages to the wound bed, angiogenesis, tissue regeneration, and remodeling. In light of this, such synchronized modulation of the healing cascade under the influence of novel hydrogel formulations appears to be the main reason for the improved burn wound repair evidenced in our previous study [14].

### 2.6. Limitations

This study used immunohistochemical analysis to observe temporal patterns of cellular and tissue responses crucial for wound healing; however, molecular quantification with quantitative data would provide greater insight into the mechanistic foundation behind the enhanced wound healing caused by the novel hydrogels. Although several immunohistochemical markers were analyzed in the current study, further research using additional markers is needed to determine the macrophage phenotypization (INOS, MANOSE), maturation and functionality of newly formed blood vessels (αSMA), and which cell populations undergo apoptosis and to what extent (TUNEL assay double staining with cell-specific markers).

Although the hydrogels were replaced daily, the dynamic mechanical interaction between the dressing and the contracting wound bed was not directly quantified. Although Gent release from the investigated formulations was previously characterized in vitro [10], the Gent concentration was not measured directly in the wound tissue or wound exudate during the present in vivo experiment. Therefore, the relationship between local Gent exposure and the observed immunohistochemical responses cannot be established. Furthermore, because both investigated formulations contained Gent and no corresponding Gent-free hydrogel controls were included, the independent contribution of Gent cannot be separated from that of the polymer matrix.

## 3. Conclusions

The principal finding of this study is that neither of the investigated hydrogels altered the physiological sequence of burn wound healing. Still, they advanced the empirical timeline for wound healing, which was evidenced by the temporal changes in macrophage recruitment, angiogenesis, cellular proliferation, and apoptosis. The novel hydrogels increased the efficiency of the regenerative process without disturbing its natural course. More potent effects were observed for the PVA/CHI/Gent hydrogel, indicating that the addition of CHI to the hydrogel formulation led to the design of a wound dressing with substantially enhanced regenerative potency. Therefore, this composite hydrogel could be further explored for its translational potential as a promising biomaterial in burn wound healing.

## 4. Materials and Methods

### 4.1. Materials

The synthesis of PVA/Gent and PVA/CHI/Gent hydrogels was carried out using the following chemicals: PVA powder (degree of hydrolysis ≥99%; Mw = 70–100 kDa; Sigma Aldrich, St. Louis, MO, USA), CHI powder (Mw = 190–310 kDa; deacetylation degree 75–85%; Sigma Aldrich, USA), and Gent sulfate solution (50 mg/mL in dH2O; Sigma Aldrich, USA). The deionized water used in the preparation of hydrogels was obtained by passing distilled water through a GenPure ultrapure water system (TKA, Stuttgart, Germany).

#### Synthesis of PVA/Gent and PVA/CHI/Gent Hydrogels

**Figure 9:**
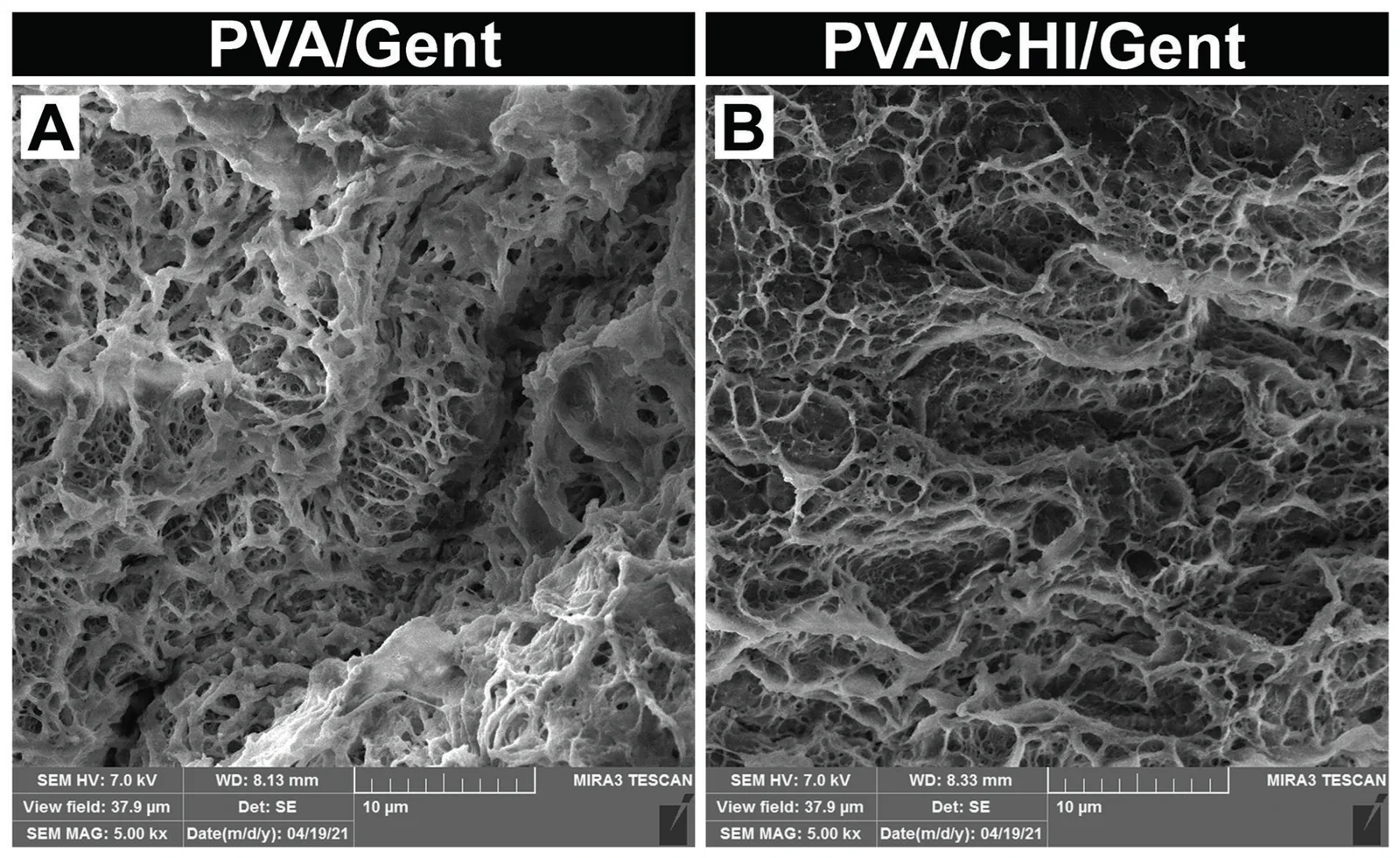
Photomicrographs of PVA/Gent (**A**) and PVA/CHI/Gent (**B**) at 5000× magnification.

Firstly, the PVA powder was dissolved in distilled water at 90 °C under magnetic stirring for 2 h to obtain a 10 wt% colloid dispersion of PVA. Additionally, for the PVA/CHI/Gent hydrogel, a 0.5 wt% CHI solution was made separately by dissolving the CHI powder in 2 vol% acetic acid (CH_3_COOH) at room temperature under continuous stirring. The prepared CHI solution was then added dropwise to the PVA solution that had cooled down to room temperature, and the resulting PVA/CHI dispersion was homogenized by stirring at room temperature for an additional 2–3 h. Both the PVA and PVA/CHI hydrogels were obtained by physical cross-linking of respective dispersions using the freezing–thawing method for five cycles. Each cycle consisted of freezing for 16 h at −18 °C, followed by thawing for 8 h at 4 °C. The formed hydrogels were then cut into discs with diameters, *d*, of 10 mm and thicknesses, *δ*, of 4 mm. To enable the loading of Gent into the hydrogels and thus achieving the final formulation used in this study, the prepared hydrogel disks were swollen in 5.0 mg/mL Gent solution at 37 °C for 48 h to ensure drug absorption. Gentamicin release from the hydrogel formulations was characterized previously by quantifying the concentration of released Gent using high-performance liquid chromatography coupled with mass spectrometry (HPLC-MS) [11]. Furthermore, the ratio between the released and initially incorporated Gent was monitored over an extended period to establish the release profile and model its diffusion behavior. Considering the daily changes of wound dressings defined in the experimental design, direct measurement of Gent concentration within the wound tissue was not conducted; therefore, local drug exposure at individual post-burn time points cannot be determined from the current data. The physicochemical, mechanical and biological characteristics of both novel hydrogel formulations, which made them candidates for preclinical testing, were shown in previous studies [11,13,14]. In our prior research, the PVA/Gent and PVA/CHI/Gent hydrogels exhibited well-defined, three-dimensional, interconnected microporous structures (Figure 9A,B), with a Young’s modulus of 1969.73 MPa measured for the PVA/CHI/Gent hydrogel. The cytotoxicity assessments demonstrated a non-cytotoxic response for both hydrogels, with cell viabilities of 93% and 92% for MRC-5 and L929 cells, respectively, for the PVA/Gent hydrogel, and 99% and 96% for MRC-5 and L929 cells, respectively, for the PVA/CHI/Gent hydrogel, as determined by an MTT assay. Moreover, both the PVA/Gent and PVA/CHI/Gent hydrogels demonstrated strong antibacterial activity, with complete elimination of both *S. aureus* and *E. coli* within 1 h of exposure [11].

### 4.2. Animal Trials

The experiment was conducted on three-month-old male Wistar rats weighing 300–330 g, obtained from the Department of Laboratory and Experimental Care and Use of Animals Unit of the Institute of Medical Research, Military Medical Academy (Belgrade, Serbia). The rats were housed in an air-conditioned animal facility under standard conditions, kept individually in polypropylene cages with a photoperiodic cycle of 12 h light: 12 h darkness, and food and water available ad libitum. All of the experimental procedures were approved by the Ethical Committee of the Faculty of Veterinary Medicine, University of Belgrade, and by the Ministry of Agriculture, Forestry, and Water Management—Veterinary Administration (decision number 323-07-04903/2022-05/1). The experiment and animal handling were carried out in accordance with the ARRIVE guidelines and the European Union’s Directive 2010/63/EU on the Protection of Animals Used for Scientific Purposes.

#### 4.2.1. Experimental Design

The induction of thermal injuries was based on the protocol developed by Tavares Pereira et al. [37]. The experimental procedure was described in our previous study [14]. In brief, animals were put under general anesthesia with intraperitoneal injections of 75 mg/kg ketamine hydrochloride (Ketamidor 10%, 100 mg/mL, RICHTER PHARMA AG, Wels, Austria) and 10 mg/kg xylazine (Xylased 2%, BIOVETA, Ivanovice na Hane, Czech Republic). Trichotomy and disinfection with povidone–iodine was performed on the skin of dorsal proximal region and burns were induced with a solid aluminum bar (10 mm diameter, mass of 51 g) preheated in boiling water (temperature of 100 °C), which was kept in contact with the skin for 15 s without added pressure. Analgesia after the procedure was administered for three consecutive days with intramuscular injections of 5 mg/kg Ketoprofen (Ketonal, at 100 mg/2 mL; SANDOZ, Basel, Switzerland). All animals survived the thermal injury induction procedure. Post-burn wound induction, the animals were randomly distributed into three experimental groups: control (Ctr) (*n* = 16), PVA/Gent-treated (*n* = 16), and PVA/CHI/Gent-treated groups (*n* = 16). In the Ctr group, the wounds were treated with gauze wetted with normal saline solution (0.9% sodium chloride), whereas in the treated groups the wounds were treated with a corresponding hydrogel formulation; additionally, all wounds were dressed daily. During the experiment, animals in all groups were monitored daily for natural behavior patterns as well as for the showing of pain using the rate grimace scale recommended by the National Center for the Replacement, Refinement & Reduction of Animals in Research. No animals showed any signs of pain and, accordingly, rescue analgesia was not needed. The survivability during the whole 21-day experimental period was 100%. No systemic antibiotic therapy was used during the experimental period. On days 3, 7, 14 and 21, four animals from each group were euthanized using 100 mg/kg Euthasol Euthanasia Solution (Produlab Pharma Production B.V., Raamsdonksveer, The Netherlands) in order to collect samples for immunohistochemical analysis.

#### 4.2.2. Tissue Processing for Immunohistochemistry

Square pieces of skin containing the whole burn were taken during sampling, and then bisected along the diameter of the circular burn. Both halves of each burn wound were fixed in 10% neutral-buffered formalin (pH 6.8), routinely processed, embedded in paraffin, sectioned at 5 μm using a rotary microtome (Cut 5062, Slee, Mainz, Germany) and mounted on special glass plates coated with a strong adhesive (Superfrost Plus Adhesion Microsope Slides, Eperida, Portsmouth, NH, USA). Tissue sections were used to determine the CD68^+^ cells (macrophage marker) (Bio RAD, Hercules, CA, USA, MCA34 1R), VEGF^+^ cells (Vascular Endothelial Growth Factor) (Invitrogen, Waltham, MA, USA, MA1-16629), ESM1^+^ blood vessels (Endothelial Cell-Specific Molecule 1) (Cloud-Clone Corp., Katy, TX, USA, PAC463Ra01), Ki-67^+^ cells (cell proliferation marker) (Abcam, Cambridge, UK, ab16667) and TUNEL^+^ cells (apoptosis marker) (Novus Biologicals, Minneapolis, MN, USA, APO-BRDU-IHC (TUNEL) Apoptosis Kit, NBP2-31164). Except for the TUNEL, for which a separate kit was used, visualization of these markers was performed using a highly sensitive and specific two-stage indirect immunohistochemical (IHC) technique, in which secondary antibodies labeled with horseradish peroxidase were directly bound to dextran polymers (Eperdia, Portsmouth, New Hampshire, USA, UltraVision Quanto Detection system HRP, TL-125-QHL). To visualize the antigen–antibody reaction, 3,30-diaminobenzidine (Eperdia, Portsmouth, NH, USA, DAB Quanto, TA-125-QHDX) was used as a chromogen. For contrasting tissue sections were stained with Mayer’s hematoxylin (Merck, Darmstadt, Germany) or methyl green, used in the TUNEL assay, and then mounted with DPX phthalate-free mounting medium (06522, Sigma-Aldrich, St. Louis, MO, USA). All immunohistochemical slides were coded prior to quantitative evaluation, and the investigators performing cell and vessel quantification were blinded to the experimental group allocation and sampling time point until completion of the measurements. Each immunohistochemical analysis was performed by a single investigator from start to finish; however, different analyses were conducted by different investigators.

#### 4.2.3. Estimation of CD68^+^, VEGF^+^, Ki67^+^ and TUNEL^+^ Cells and Relative Density of Blood Vessels

The estimation of CD68^+^, VEGF^+^, Ki67^+^ and TUNEL^+^ cells was performed in the dermal region of burn wound samples taken on days 3, 7, 14 and 21 after burn induction. Positive cells were counted on 10 non-overlapping fields of view per section using a high-power objective lens (40×). From each sample, three sections separated by 50 µm intervals were analyzed in this manner. Fields of view were distributed as follows: 4 fields of view at the edges of the wound (2 per each edge) and 6 fields of view from the central part of the wound. In each location, one field of view was positioned near the surface of the wound, and the other was positioned beneath in the deeper region of the dermis. The counted cells were then expressed as number of positive cells per mm^2^ of dermis surface area. Additionally, the Ki67^+^ and TUNEL^+^ cells were assessed in the newly formed epidermis in the burn wound samples taken on post-burn days 14 and 21. Positive cells were counted on 10 non-overlapping fields of view per section using a high-power objective lens (40×). From each sample, two sections separated by 50 µm intervals were analyzed in this manner. In each section, 5 fields of view were analyzed in the epithelial tongues on each side of the wound, with the first field positioned in the newly formed epithelium closest to the intact skin, and the fifth field positioned closest to the migrating front of keratinocyte division. The counted cells were then expressed as number of positive cells per 100 keratinocytes.

The ESM1^+^ blood vessels were assessed on 30 fields of view per sample using a high-power objective lens (40×). The fields of view were chosen and distributed in the same manner used to analyze the CD68^+^, VEGF^+^, Ki67^+^ and TUNEL^+^ cells in the dermis. Only the surface of blood vessels showing positive endothelial cells was measured using the closed polygon function from the measurement toolbox in cellSens Entry software v.1.13 (Olympus, Tokyo, Japan). The relative density of blood vessels (%) was then calculated as a percentage of dermal surface covered by ESM1^+^ blood vessels in the burn wound samples taken at all experimental time periods.

All microscopic examinations and measurements were performed using an Olympus CX31 microscope equipped with cellSens Entry software v.1.13 (Olympus, Tokyo, Japan). Representative photomicrographs were captured using an Olympus UC50 digital camera (Olympus, Tokyo, Japan).

### 4.3. Statistical Analysis

All collected data were analyzed in GraphPad Prism 9 software (GraphPad, San Diego, CA, USA). The normality of data distribution was assessed using the Shapiro–Wilk test and comparisons among experimental groups were performed by Kruskal–Wallis test followed by Dunn’s post hoc test. Differences were considered statistically significant at significance levels of *p* < 0.05, *p* < 0.01, *p* < 0.001, and *p* < 0.0001.

## Data Availability

The raw data supporting the conclusions of this article will be made available by the authors on request.
